# Biostatistics: Facing the Interpretation of 2 × 2 Tables

**DOI:** 10.5334/jbr-btr.1399

**Published:** 2017-12-16

**Authors:** Adelin Albert

**Affiliations:** 1 ULG, BE

**Keywords:** Agreement coefficient, Binary variable, Comparison of proportions, Diagnostic test, Odds ratio

## Abstract

There are multiple ways in which 2 × 2 tables arise in clinical research. Different facets of 2 × 2 tables can be identified which require appropriate statistical analysis and interpretation. This paper presents a brief overview of such tables.

## Introduction

In statistics, 2 × 2 tables are generally obtained by cross-classifying data from two binary variables; one variable will represent the rows of the table and the other the columns. For example, if gender (male, female) and smoking (no, yes) are being recorded for n subjects, data will best be summarized by a 2 × 2 table displaying gender against smoking. Numbers in cells of the table are counts, not measurements. Thus, in the example, the cells will contain the number of male smokers and nonsmokers and the number of female smokers and nonsmokers, respectively. Contingency (or count) 2 × 2 tables are among the most basic concepts taught in any elementary course in statistics, along with the mean, the standard deviation, and the correlation [1]. In the literature, their use is pervasive, particularly when it comes to comparing two proportions. But there are many different situations in which 2 × 2 tables arise, depending on the problem at hand, the way patients were selected, and the research context. Several distinct facets of 2 × 2 tables are evident in the literature. Six of them will be briefly described here. They should help clinicians discern which 2 × 2 table he or she is facing, use the appropriate statistical test, and provide the correct interpretation.

## Notation

For clarity, we denote *X*, the row variable, and *Y*, the column variable, but this notation can be reversed. Both variables are binary and take values (e.g., 0 and 1). It is also convenient to denote by *a, b, c*, and *d* the number of observations (clockwise) in the 4 cells of the 2 × 2 table (see Table 1). In this table, the row totals, (*a* + *b*) and (*c* + *d*), and the column totals, (*a* + *c*) and (*b* + *d*), are called the *margins* because they define the marginal distributions of *X* and *Y*, respectively. The sum of all cells is the grand total *n*.

**Table 1:** General Representation of a 2 × 2 Contingency Table.


Variable *X*	Variable *Y*		Total
	0	1	

0	*a*	*b*	*a* + *b*
1	*c*	*d*	*c* + *d*
Total	*a* + *c*	*b* + *d*	*n*

From a statistical sampling standpoint, there are only three ways to establish a 2 × 2 contingency table: (i) the row margins (*a* + *b*) and (*c* + *d*) are fixed, in which case the column margins are observed and percentages can only be calculated horizontally; (ii) the column margins (*a* + *c*) and (*b* + *d*) are fixed, in which case the row margins are observed and percentages can only be calculated vertically; or (iii) the grand total *n* is fixed, in which case all elements and margins of the table are observed and percentages can be calculated by row, by column, or globally. Thus, when facing a 2 × 2 table, it is important to know how the table was established.

## Case 1: Comparing Two Independent Proportions

This is the most familiar case. Smoking (No/Yes) was assessed in a sample of 1,262 high school boys and in a separate sample of 1,132 high school girls of the province of Luxembourg (data not published). Data are displayed in Table 2. In this table, column margins were fixed, and all other numbers were observed. Thus, percentages can only be derived vertically. The proportion of smokers among boys is 389/1,262 (30.8%) and among girls 372/1,132 (32.9%). Are these two proportions statistically different?

**Table 2:** Smoking in High School Boys and Girls in the Province of Luxembourg (Belgium).


Smoking	Gender		Total
	Boys	Girls	

No	873	730	1,633
Yes	389	372	761
Total	1,262	1,132	2,394

To test the null hypothesis of equal proportions of smokers in high school boys and girls, we use the “homogeneity test” by calculating the renowned chi-squared test on 1 degree of freedom (\chi _{(1)}^2) in which all terms can be found in the 2 × 2 contingency table:

\chi_{\left(1 \right)}^2 = \,\,\,\frac{{\left({ad - bc} \right)^{2}n}}{{\left({a + b} \right)\left({c + d} \right)\left({a + c} \right)\left({b + d} \right)}}

Thus:

\chi_{\left(1 \right)}^2 = \,\,\frac{{{{\left({873\,\, \times \,\,372 - 730\,\, \times \,\,389} \right)}^2} \times \,\,2394}}{{1633\,\, \times \,\,761\,\, \times \,\,1262\,\, \times \,\,1132}}\,\, = 1.14

Since the associated *p*-value (*p* = 0.29) is not significant, we conclude that smoking is comparable in boys and girls.

## Case 2: Testing the Correlation between Two Binary Outcomes

This case is often confounded with the homogeneity test above. Postoperative nausea (No/Yes) and vomiting (No/Yes) were recorded in 671 surgical patients [2]. Data are displayed in Table 3. In this table, only the grand total sample size *n* was fixed so that all other numbers were observed. Is there an association (correlation) between nausea and vomiting? The null hypothesis of no correlation between the two symptoms can be assessed by the “independence test” by computing a chi-squared test similar to the homogeneity test above (whence the confusion).

**Table 3:** Postoperative Nausea and Vomiting in 671 Surgical Patients.


Nausea	Vomiting		Total
	No	Yes	

No	532	13	545
Yes	73	53	126
Total	605	66	671

Applying the formula above (see Case 1), we get:

\chi_{\left(1 \right)}^2 = \,\,\frac{{{{\left({532\,\, \times \,\,53\,\, - 13\,\, \times \,\,73} \right)}^2}\, \times \,\,671}}{{545\,\, \times \,\,126\,\, \times \,\,605\,\, \times \,\,66}} = 181.7

The large chi-squared value evidenced a highly significant association between postoperative nausea and vomiting in surgical patients (*p* < 0.0001), and the hypothesis of independence between the two symptoms was rejected. In other words, there is a strong dependency between them. Note that the correlation between nausea and vomiting can easily be derived from the chi-squared test by calculating the expression r = \sqrt {{\chi^{2}}_{(1)}\,/\,n} = \sqrt {181.7\,/\,671} = 0.52.

## Case 3: Comparing Two Paired (Matched) Proportions

In contrast to the homogeneity test, the McNemar test [3] allows the comparison of two paired proportions obtained on the same subjects or on matched individuals. Data reported in Table 4 concern the distance walked (≤500 m or >500 m) before and after surgery by 156 patients suffering from degenerative lumbar stenosis with neurogenic intermittent claudication (unpublished data). In this table, the grand total *n* was fixed so that all other numbers in the table were observed.

**Table 4:** Walking Distance before and after Surgery of 156 Patients Suffering from Degenerative Lumbar Stenosis with Neurogenic Intermittent Claudication.


Before Surgery	After Surgery		Total
	≤500 m	>500 m	

≤500 m	56	37	93
>500 m	20	43	63
Total	76	80	156

The proportion of patients who walked more than 500 m before surgery was 63/156 (40.4%), while the proportion after surgery was 80/156 (51.3%). Are these two proportions significantly different? The homogeneity test cannot be used because the two proportions were obtained on the same 156 patients; they are correlated. The null hypothesis of equal proportions is tested by the McNemar chi-squared test on 1 degree of freedom:

\chi_{\left(1 \right)}^2 = \frac{{\left({b\,\, - \,\,c} \right)}^{2}}{{b\,\, + \,\,c}}

Using data in Table 4, we get:

\chi_{\left(1 \right)}^2 = \frac{{\left({37\,\, - \,\,20} \right)^2}}{{37\,\, + \,\,20}} = 5.07\,\left({p\,\, = \,\,0.024} \right)

This shows a significant difference between the two proportions. In other terms, the surgical treatment did improve the walking distance of patients.

## Case 4: Assessing the Degree of Agreement between Two Raters

The degree of agreement between two raters or methods can best be measured by the Cohen kappa (κ) coefficient [4]. As an illustration, data in Table 5 were obtained by cross-classifying the diagnosis (benign or malignant) of 187 suspected tumors made by 2D mammography and 3D tomosynthesis (data not published). Readings were made by a senior radiologist. Once again, the grand total *n* was fixed, and all numbers in the table were observed.

**Table 5:** Diagnosis of 187 Suspected Tumors by 2D Mammography and 3D Tomosynthesis.


Mammography	Tomosynthesis		Total
	Benign	Malignant	

Benign	54	68	122
Malignant	14	51	65
Total	68	119	187

One may think here of the McNemar test as in Case 3; indeed, the proportion of malignancy was 65/187 (34.8%) for mammography and 119/187 (63.6%) for tomosynthesis, and the chi-squared test was equal to \chi_{(1)}^{2} = \frac{(68 - 14)^{2}} {68 + 14} = 35.6\ (p\ <\ 0.0001), indicating a highly significant difference between the two proportions. In other terms, the two radiological methods do not give the same outcomes; this tends to indicate that they do not really agree with each other, which can best be demonstrated by computing Cohen κ coefficient as follows.

Let *p*_o_ = (*a* + *d*)/*n* the observed proportion of agreements between the two raters. From data in Table 5, *p*_o_ = (54 + 51)/187 = 0.561. Next, compute the expected proportion of agreements due to chance only (as if the two raters were to decide randomly and independently of each other). Denote by *p*_e_ = [(*a* + *b*).(*a* + *c*) + (*c* + *d*).(*b* + *d*)]/*n*^2^ this proportion. In our example, we have. *p*_e_ = [122 × 68 + 65 × 119]/(187)^2^ = 0.458. Then, Cohen kappa coefficient writes:

\kappa = \frac{{{p_o}\, - \,\,{p_e}}}{{1\,\, - \,\,{p_e}}} = \frac{{0.561\,\, - \,\,0.458}}{{1\,\, - \,\,0.458}} = 0.19.

The closer κ is to 1, the better the agreement between the two raters. The value of 0.19 is quite low, indicating poor agreement between the two diagnostic methods, hence confirming the highly significant McNemar test.

## Case 5: Measuring the Diagnostic Value of a Clinical Test

In medical practice, assessing the diagnostic (prognostic) ability of a clinical (biological, radiological) test is often required [5]. This is traditionally done by using concepts such as diagnostic specificity and sensitivity and positive (negative) predictive value. In this context, the row variable *X* is the clinical test (*T*) to be assessed (negative, positive) and the column variable *Y* the disease (*D*) to be diagnosed (absent, present). As an example, consider the Folin-Wu colorimetric test to assay blood glucose. Remein and Wilkerson [6] applied this test to 510 presumably healthy subjects and to 70 diabetic patients. Data are given in Table 6. In this table, column margins were fixed and all other numbers were observed. Thus, percentages can only be derived vertically.

**Table 6:** Diagnostic Ability of Folin-Wu Test for Diabetes.


Folin-Wu Test	Diabetes		Total
	Absent	Present	

Negative	461	14	475
Positive	49	56	105
Total	510	70	580

As in Case 1, we could compute the proportions of positive tests in healthy and diabetic subjects and compare them by a chi-square test, but this is clearly not the purpose here. Instead, we shall investigate how the laboratory test performs in diseased and nondiseased subjects.

We would expect the clinical test to be mostly negative in healthy individuals. This can be measured by the specificity of the test *SP* = *a*/(*a* + *c*), the proportion of negative results in healthy (nondiseased) subjects. In contrast, we would expect the clinical test to be predominantly positive in diseased subjects. This can be measured by the sensitivity of the test *SE* = *d*/(*b* + *d*), the proportion of positive results in diseased subjects. The overall efficacy of the test which combines specificity and sensitivity writes *EF* = (*SP* + *SE*)/2. The specificity is also called the *true negative rate* (*TN*) and the sensitivity the *true positive rate* (*TP*). The *false positive rate* (*FP* = 1 – *TN*) and the *false negative rate* (*FN* = 1 – *TP*) are also familiar clinical terms. Applying these concepts to the Folin-Wu test data in Table 6, we have *SP* = 461/510 = 0.904 (90.4%) and *SE* = 56/70 = 0.80 (80%) so that the efficacy is *EF* = (0.904 + 0.80)/2 = 0.852 (85.2%). Further, the false positive and negative rates are *FP* = 1 – 0.904 = 0.096 (9.6%) and *FN* = 1 – 0.800 = 0.20 (20.0%).

The positive predictive value (*PPV*) of the test which measures the proportion (probability) of diseased subjects among those with a positive test cannot simply be derived from the table or from the specificity and sensitivity. Indeed, as column totals have been fixed, numbers cannot be divided horizontally as indicated before; thus, *PPV* is not equal to *d*/(*c* + *d*). To compute *PPV*, one needs to know the prevalence (frequency) *π* of the disease in the population. Then, Bayes theorem is used:

PPV = \,\,\frac{{\pi SE}}{{\pi SE + \,\,\left({1\,\, - \,\,\pi} \right)\left({1\,\, - \,SP} \right)}}

For the Folin-Wu study, assuming a prevalence of diabetes in the population of 6% (*π* = 0.06), the VPP turns out to be 34.7%:

VPP = \frac{{0.06\,\, \times \,\,0.80}}{{0.06\,\, \times \,\,0.80\,\, + \,\,\left({1\,\, - \,\,0.06} \right)\,\, \times \,\,\left({1 - \,\,0.904} \right)}} = 0.347.

In other terms, when the Folin-Wu colorimetric test is positive, the subject has a 34.7% chance of having diabetes, which is substantially higher than the expected 6% before the test was performed. Similarly, the negative predictive value (*NPV*) is defined as the proportion of subjects free of the disease among those with a negative test. It should be emphasized that *NPV* is not given by *a*/(*a* + *b*), but rather by the formula:

NPV = \,\,\frac{{\left({1 - \pi } \right)SP}}{{\left({1 - \pi} \right)SP + \,\,\pi \left({1 - SE} \right)}}

For the Folin-Wu data, we have:

NPV = \frac{{\left({1\,\, - \,\,0.06} \right)\,\, \times \,\,0.904}}{{\left({1\,\, - \,\,0.06} \right)\,\, \times \,\,0.904\,\, + \,\,0.06\,\, \times \,\,\left({1\,\, - \,\,0.80} \right)}} = 0.948\left({94.8\%} \right)

Thus, when the Folin-Wu test is negative, diabetes can almost surely be excluded.

Returning to the diagnosis of suspected tumors by 2D mammography and 3D tomosynthesis (readings by a senior radiologist), the 156 tumors were also analyzed by a pathologist (gold standard). It turned out that the specificity and sensitivity were equal to 78% and 36% (*EF* = 40%), respectively, for mammography and 83% and 69% (*EF* = 70%), respectively, for tomosynthesis, emphasizing the better diagnostic ability of the latter technique.

## Case 6: Measuring the Association between a Risk Factor and a Disease

One of the main objectives of epidemiological studies is to assess the association between a risk factor and a disease by means of 2 × 2 tables. This gives rise to the renowned notions of relative risk (*RR*) and odds ratio (*OR*). In this context, the row variable *X* is the risk factor (*F*) to which subjects are exposed, or not, and the column variable *Y* is the disease (*D*) which can develop, or not, in subjects.

As an example, consider the retrospective study of Hiller and Kahn [7], who looked at the association between diabetes (the risk factor) and eye cataract (the disease) in 607 patients with cataract and in 2,011 patients free of cataract. Data are summarized in Table 7. Here too column margins (totals) have been fixed, and the other numbers have been observed. This looks similar to Case 1, where proportions were compared in two different groups. The present goal, however, is to measure the association between diabetes and cataract, specifically to assess diabetes as a potential risk factor for developing cataract. This example also shows that a disease (diabetes) can become a risk factor for another disease (cataract).

**Table 7:** Association between Diabetes and Eye Cataract in Subjects Aged 50–69 Years.


Diabetes	Cataract		Total
	Present	Absent	

Yes (Exposed)	55	84	139
No (Nonexposed)	552	1927	2,479
Total	607	2,011	2,618

In such studies, the association between the risk factor (*F*) and the disease (*D*) is quantified by the odds ratio defined as *OR* = *ad/bc*, a definition to be found in any textbook in epidemiology [8,9]. A value *OR* > 1 indicates a positive association between the risk factor and the disease (increased risk), while when *OR* < 1, the association is negative (decreased risk). When *OR* = 1, there is no association between risk factor and disease.

Data in Table 7 reveal that the risk of cataract is more than doubled in diabetic patients compared to nondiabetic ones:

OR\,\, = \,\,\frac{{55\,\, \times \,\,1,927}}{{84\,\, \times \,\,552}}\,\, = \,\,2.29.

The odds ratio is significantly different from 1 as confirmed by the 95% confidence interval (95% CI: 1.6–3.3), but also by the chi-squared homogeneity test described in Case 1 (*p* < 0.0001).

Odds ratios have become very popular to measure the association between a risk factor and a disease, even in a clinical environment. They are also used in cross-sectional, prospective, and cohort studies, where normally the relative risk (RR) should be preferred. They are easily derived and generalized by (multivariate) logistic regression analysis when it comes to studying the association between several risk factors for a single disease [10,11].

## Discussion

Clinicians and researchers are regularly faced with 2 × 2 contingency tables, particularly when analyzing small datasets or large databases containing binary data. Although simple at first glance, their interpretation can sometimes become difficult. We have insisted on the way 2 × 2 tables were established. Were row or column margins fixed or was the grand total fixed? This is particularly important when it comes to calculating percentages; dividing cell numbers by totals must be done with caution. A remarkable example is the calculation of positive predictive values.

Two-by-two tables arise in various situations, as we have seen, and the way to analyze the data should be done cautiously. For instance, when comparing two proportions from distinct groups (Case 1: column margins fixed), it makes no sense to calculate the correlation between the two binary variables. This can only be done when both variables have been observed together (Case 2: grand total fixed). Thus, for the comparison of smoking in male and female teenagers, we cannot conclude the independence between smoking and gender nor calculate a correlation coefficient. By contrast, when fixing the grand total *n* (Case 2), we can compare the two column or row proportions without restrictions. For instance, in Table 3, we can assert that the proportion of patients with vomiting was higher in patients with nausea (53/126, 42.1%) than in patients without nausea (13/545, 2.4%).

The distinction between independent proportions (Case 1) and paired proportions (Case 3) is also essential. Applying the homogeneity test where the McNemar test is requested can lead to fallacious conclusions because the proportions to be compared are not the same. As an illustration, the homogeneity test applied to data in Table 4 yields a highly significant value \chi_{(1)}^2\, = \,\,12.2 (*p* = 0.0005), but it shows that the proportion of patients walking >500 m after surgery is higher among those who walked >500 m (43/63, 68.3%) than those who walked ≤500 m before surgery (37/93, 39.8%); this comparison gives only a partial view of the surgical efficacy.

We already mentioned the relationship between Cohen kappa coefficient (Case 4, agreement between raters) and the McNemar test (Case 3); in both tables, the grand total was fixed. A significant McNemar test corresponds to a κ coefficient significantly different from 0, but it does not necessarily mean that there is a high degree of agreement between the two raters, particularly when the sample size is large. In relation to the assessment of the diagnostic capacity of a clinical test (Case 5), it should be emphasized again that the *PPV* cannot simply be derived from the 2 × 2 table because, in general, the columns are fixed, and dividing can only be done vertically. Therefore, the prevalence (proportion of diseased subjects in the population) needs to be specified separately. In some rare situations where the grand total *n* is fixed, the prevalence can be estimated by *π* = (*b* + *d*)/*n*, so that *PPV* = *d*/(*c* + *d*) directly from the 2 × 2 table.

Finally, for measuring the association between a risk factor and a disease (Case 6), we only mentioned the odds ratio, a widely used indicator in epidemiological and clinical studies. In prospective or cohort studies, however, where a sample of subjects exposed to the risk factor and a separate sample of nonexposed subjects are followed up over time and the occurrence of the disease recorded (row margins are fixed rather than column margins), *RR* should be preferred to *OR*. By definition, the relative risk is the ratio of the incidence rate of the disease in exposed and nonexposed subjects, specifically, *RR* = [*a*/(*a* + *b*)]/[*c*/(*c* + *d*)]. Its interpretation is similar to that of *OR*. In cross-sectional studies in which the risks factor and the disease are observed simultaneously, the relative risk is the ratio of the prevalence rate (not the incidence rate) of the disease in exposed and nonexposed subjects, but the formula remains the same.

In conclusion, 2 × 2 tables are common place in the medical literature and one of the first summary statistics taught in any basic textbook. When facing such a table, ask yourself which totals (margins) are fixed (row, column, or grand total); calculate the appropriate percentages; perform the adequate statistical test; and provide the best interpretation of the data.
